# Development of Phase Detection Schemes Based on Surface Plasmon Resonance Using Interferometry

**DOI:** 10.3390/s140915914

**Published:** 2014-08-28

**Authors:** Muhammad Kashif, Ahmad Ashrif A. Bakar, Norhana Arsad, Sahbudin Shaari

**Affiliations:** Department of Electrical, Electronic and Systems Engineering, Faculty of Engineering and Built Environment, Universiti Kebangsaan Malaysia, 43600 UKM Bangi, Selangor, Malaysia; E-Mails: kaashpk@bzu.edu.pk (M.K.); norhana@eng.ukm.my (N.A.); sahbudin@eng.ukm.my (S.S.)

**Keywords:** surface plasmon resonance, sensors, interferometry, Fabry-Perot, Mach-Zehnder, Michelson

## Abstract

Surface plasmon resonance (SPR) is a novel optical sensing technique with a unique ability to monitor molecular binding in real-time for biological and chemical sensor applications. Interferometry is an excellent tool for accurate measurement of SPR changes, the measurement and comparison is made for the sensitivity, dynamic range and resolution of the different analytes using interferometry techniques. SPR interferometry can also employ phase detection in addition to the amplitude of the reflected light wave, and the phase changes more rapidly compared with other approaches, *i.e.*, intensity, angle and wavelength. Therefore, the SPR phase interferometer offers the advantages of spatial phase resolution and high sensitivity. This work discusses the advancements in interferometric SPR methods to measure the phase shifts due to refractive index changes. The main application areas of SPR sensors are demonstrated, *i.e.*, the Fabry-Perot interferometer, Michelson interferometer and Mach-Zehnder interferometer, with different configurations. The three interferometers are discussed in detail, and solutions are suggested to enhance the performance parameters that will aid in future biological and chemical sensors.

## 1. Introduction

A significant research has been done in the area of optical sensors for the measurement of chemical and biological samples. A large number of methods have been used for biosensing and chemical sensing, *i.e.*, ellipsometric porosimetry [1], *in*-*situ* ellipsometry [2], imaging ellipsometry [3], phosphorescence spectroscopy [4], luminescence spectroscopy [5], fluorescence spectroscopy [6] and Raman spectroscopy [7] as well as the spectroscopy, interferometry and surface plasmon resonance SPR [8]. The given sample amount is measured by sensors through fluorescence, absorbance and *n* properties of analyte molecules. The SPR process can be observed at metal interfaces as well [9]. Therefore, SPR sensing has received increasing attention from the scientific community and significant efforts are done in the field of SPR biosensing. The chemical and biological samples are measured by using new SPR techniques development has been reported together with their shortcomings and solutions. [10]. The SPR technique is used for measuring kinetic rates of reactions (rates of complex formation and dissociation) [11], affinity (strength of binding) [12], concentration of purified protein or protein in a complex mixture [13], binding partner identification [14], biomolecular complex formation [15], quantification of antibiotics in honey [16], screening for milk testing [17], veterinary drug residues [18], detection of antibodies to salmonella in meat [19] and the presence of genetically modified organisms [20]. The SPR setup is controlled and evaluated via software, and no fluorescent or radioactive labels are required. This technique offers definite advantages in speed and throughput with safe and easy use [21–23].

Other available biosensors display different drawbacks compared with those of SPR biosensors, e.g., impedance spectroscopy (IS) uses complex digital signal processing for measurement of impedance variations of a unit [24]. Micro-cantilever biosensors (MCBs) are nanomechanical sensors have emerged as a label-free detection method, but this approach can be negatively affected by humidity and pressure [25]. Carbon nanotubes (CNTs) are used in biosensors due to change in electrical (the ability to vary the electrical property) and electrochemical properties (metallic to semiconductor materials) [26]. However, control of the diameter and signal processing of these sensors is difficult. Fluorescence biosensors have a lot of applications in the area of drugs, DNA sequencing, purification and cloning. However, this technique requires fluorescent labeling apart from native fluorescence [27]. Chemi-luminescence (CL) is a substitute method for on-chip detection of biomolecules as compared to fluorescence. However, CL performance is degraded due to the factors such as temperature, pH of the solvent etc [28]. An acoustic wave is generated by surface acoustic wave (SAW) sensor that travels on the piezoelectric crystal surface to produce sensitive surface [29]. One disadvantage of SAW is that Rayleigh waves are surface-normal waves that are poorly suited for liquid sensing. A quartz crystal microbalance (QCM) generates an electrical signal produced by piezoelectric gold plated device. The shortcoming of QCM is that the measure of resonant frequency shift is the only parameter available and has restricted this method to mass sensing of thin and rigid films [30].

Other instrumental schemes use evanescent waves to excite surface plasmons (SP). The sensitivity of SPR prism coupler is better as compared to SPR-grating-based sensors [31]. In the Otto configuration, which uses the attenuated total reflectance, the fixed distance between the right angle prism and the metal film at a given value of sample can cause issues [32]. Fiber optic SPR sensors have attained considerable attention recently due to the construction of miniaturized sensors. In fiber-based SPR sensors only few wavelength scans are possible due to almost fixed incident angle problem. Another issue is the limited wavelength range of the optical fiber [33]. To observe the interference phase changes the experiment must be performed continuously in order to have thickness and n values in case of dual polarization interferometry. The features of resonant waveguide grating (RWG) biosensors are widely used in drug detection for monitoring the changes in cell binding of live cells in real-time [34]. A partial penetration of an evanescent wave that cause the miscalculations in evaluating certain cells due to the large size of those cells and therefore, observations can only be conducted on a cell limited portion [11].

This review discusses the basic theory of SPR phase detection via interferometry. Three prominent interferometers are discussed for SPR phase detection: Fabry-Perot, Mach-Zehnder and Michelson interferometers. Another comparison is drawn to demonstrate that the phase is highly sensitive to *n* changes compared with wavelength, angle and intensity. The SPR phase has become a prominent technique in the optical biosensor field due to its increased sensitivity. The article provides a summary of the current applications of this technique and aids researchers in this area toward the development of efficient phase-sensitive SPR sensors for detection of small molecules with greater output.

## 2. Theory of SPR Phase Detection

Research is focused to determine the phenomenon of energy transmission from a single plane light wave to the SPW that travels along an interface of metal and dielectric with determination of the reflection coefficient of the incident light. The steep variation in the reflection curve is usually determined to get information about the properties of the metal dielectric interface by using either an angular or wavelength SPR dispersion technique [35]. However, only p-polarization of the electromagnetic field can cause the evanescent waves that produce a change in each electric field reflection, and the vector direction can be measured by the amplitude ratio of its p- and s-components. The s-polarized and p-polarized light act as the reference and measurement beams, respectively [36]. These detection techniques are commonly used for the development of environmental, chemical and biological small amount detection. To increase the sensing performance the higher detection sensitivity is required. Therefore the high sensitivity for SPR sensors can be achieved by measuring the sharp phase variation during SPR occurs has been compromised due to complexity [37].

**Figure 1.**
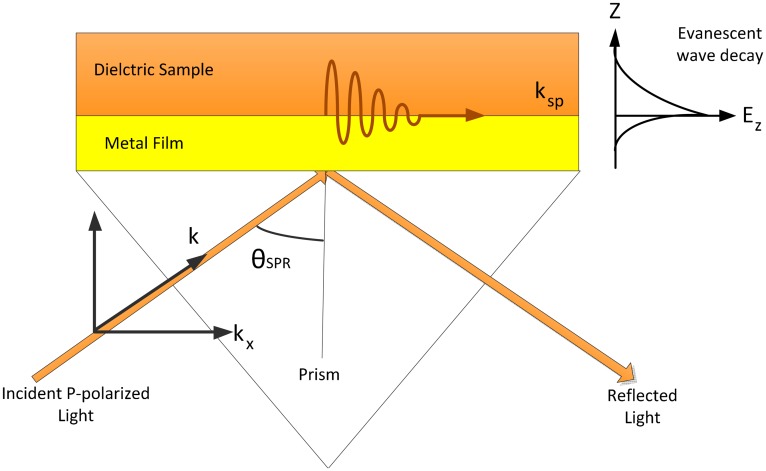
Schematic of SPs propagating in the x-direction. The exponential behaviors of the evanescent field is indicated on the right.

It is appropriate to discuss the mathematical description of the evanescent wave, which plays a key role in the concept of SPR sensing. When an electromagnetic plane wave propagates in a medium with refractive index, *n*, it can be expressed mathematically as a function of an electric field (E). The evanescent wave travels parallel to the interface of the metal and sample medium with an electric field amplitude that decays exponentially along the y-direction, given by,

(1) $$E=E_{0}e^{-k_{y}y}\text{exp}(j\omega t-jk_{x}x)$$

where $E_{0}e^{–k_{y}y}$ is the amplitude of the electric field, *ω* is the angular frequency, *x* is the position vector and *k_x_* is the wave vector. The SPs are excited by the energy of the optical light beam, which quickly dissipates and decays in the form of heat during propagation as shown in Figure 1. Therefore, the wave vector coupling condition must satisfy this relationship:

(2) $$k_{x}=\frac{\omega }{c}\sqrt{{ɛ}_{\text{prism}}}\sin\theta$$

(3) $$k_{sp}=\frac{\omega }{c}\sqrt{\frac{{ɛ}_{\text{metal}}{ɛ}_{\text{dielectric}}}{{ɛ}_{\text{metal}}+{ɛ}_{\text{dielectric}}}}$$

(4) $$k_{x}=k_{sp}$$

(5) $$n_{\text{glass}}\sin\theta =\frac{\omega }{c}\sqrt{\frac{{ɛ}_{\text{metal}}{ɛ}_{\text{dielectric}}}{{ɛ}_{\text{metal}}+{ɛ}_{\text{dielectric}}}}$$

Thus, the electromagnetic wave vector (*k_x_*) must be equal to the charge density wave vector (*k_sp_*) to perform SPR, where *ω* is angular frequency, *c* is the speed of light, *ε_metal_* is the dielectric constant of the metal, *ε_dielectric_* is the dielectric of the sample and *ε_prism_* is the dielectric constant of the glass prism [38]. Certain properties of SPs, *i.e.*, enhancement, phase jump during SP excitation and SP coherence length, are related to SPR sensor applications [39].

Enormous field enhancement occurs during SPR, and SPR dip is highly sensitive to the changes in the sample *n* of the dielectric at the interface [40]. At an optimal film thickness for SPR, the phase of light can cause a sharp dip in the angular dependence of the phase on the p-polarized light under SPR. The main light beam is divided into two portions: one is a “probe” beam, and the second is a “reference” beam used for comparison with the s-polarized portion of the main beam. [41]. The theoretical calculations show 10^2^ to 10^3^ times greater sensitivity than the conventional setup for the phase measurement [41]. Because the dispersion of the SPs is highly sensitive to the *n* of the adjacent medium of the metallic film, the SPR technique is used for highly sensitive sensors, *i.e.*, affinity biosensors. The affinity SPR biosensors are used for the detection of biological molecules at present that are highly sensitive. Additionally, the phase changes are modulated due to reflection in SPR. The phase variation of the reflected light beam occurs when SPs propagate on the metal film surface [42].

Therefore, the phase shifts Δ*ϕ* due to interference can be observed by spatial displacement of the light beam. The measurement of the phase shift Δ*ϕ_max_* for SPR conditions produces a change in the effective *n* of a medium by which the phase derivative Δ*ϕ*/Δ*n* can be measured. The phase suffers a rather sharp jump of Δ*ϕ_max_* < 2*π* at a narrow range of angle Δ*θ_ph_* close to the SPR dip, whereas the changes in the intensity Δ*θ_int_* are significantly wider Δ*θ_int_* ≫ Δ*θ_ph_* within the angular span. Such a change in the SPR interference pattern by approximately one fringe is equal to the phase shift Δ*ϕ* = 2*π* [43].

**Figure 2.**
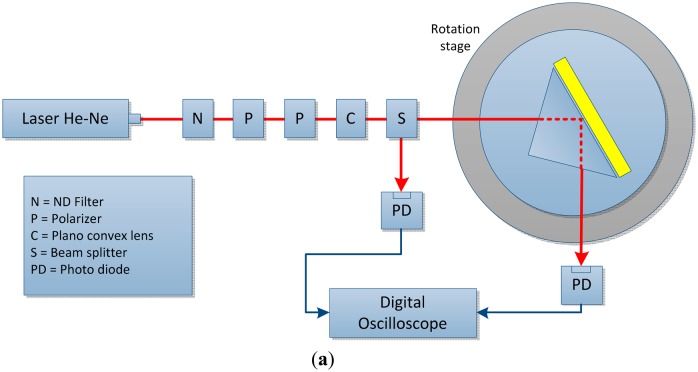

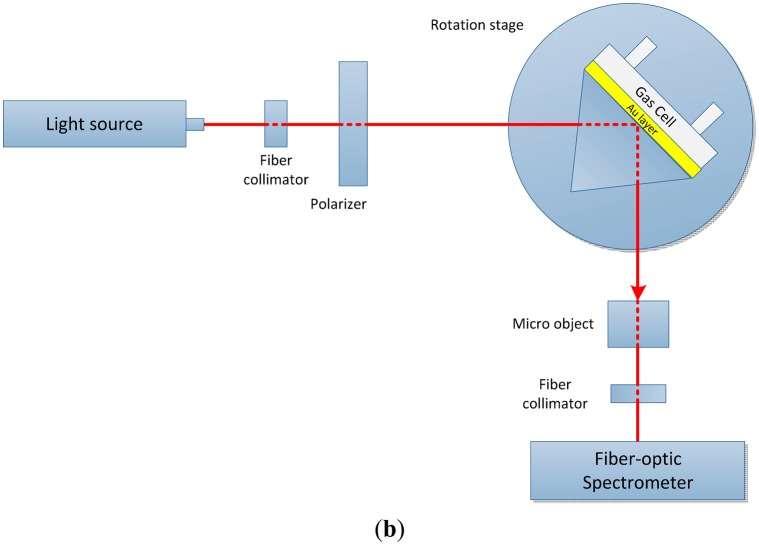
(**a**) Experimental setup for an angular interrogation SPR sensing system; (**b**) Experimental setup for wavelength interrogation.

In spectroscopy-based SP sensors, the wavelength or angular spectrum of a light wave coupled to a SP is determined, and the output of the sensor is linked to a change in the wavelength or the angular position of the SPR dip [12]. For an SPR sensor with angle interrogation, the SPs are excited on the gold film surface using a laser as shown in Figure 2a. A plano-convex lens is used to collimate the light on the SPR surface, and a *θ* − *2θ* motorized rotation stage is used to connect two-axis stepping motor drive controllers. Several lenses and filters are used to produce high sensitivity [44,45]. When SPR occurs, the reflected light intensity is the minimum determined with a photodetector. In the case of the wavelength interrogation technique, a polarizer is used as an analyzer for light entering the spectrometer and is mounted on a rotation stage as shown in Figure 2b.

An iris diaphragm is used to filter out the radiation field [46]. The output light is inputted to the spectrometer through a microscope objective via a coupling fiber. The wavelength linked to the dip in the SPR transmission spectra is the SPR wavelength, and the spectral characteristics of the waveguide biosensor are measured by the spectrometer [47,48].

The main performance parameters for an SPR biosensor include sensitivity, signal-to-noise ratio (SNR), accuracy, precision, repeatability and minimum detection limit are described in Table 1.

## 3. Introduction to Interferometry

Interferometry is a popular analytical technique used in optics for measurement of *n* changes, small displacements and surface irregularities as well as in spectroscopy for analysis of light absorption or transmission parameters associated with a substance [54]. The principle of superposition is used in interferometry to intermix the waves in such a way that produces the combinational output to get some useful properties that identify the original states of the waves. The biochemical reaction information can be deduced by obtaining the phase change from the interference pattern [55]. Three basic types of interferometers that are commonly used in SPR interferometry are the Fabry-Perot interferometer (FPI), Mach-Zehnder interferometer (MZI), and Michelson interferometer types.

A FPI normally consists of a transparent plate with two highly reflective surfaces and multiple beam interference. The transmission spectrum is a function of the wavelength that shows peaks of large transmission. The light is partially transmitted through the interferometer, and the remaining portion of the light is reflected back [56]. The Fabry-Perot setup can be used to determine the *n*, temperature, wavelength and control light wavelengths or to measure geometrical properties [57,58]. The geometric features are defined by many properties, *i.e.*, *n*, thickness and beam direction.

**Figure 3.**
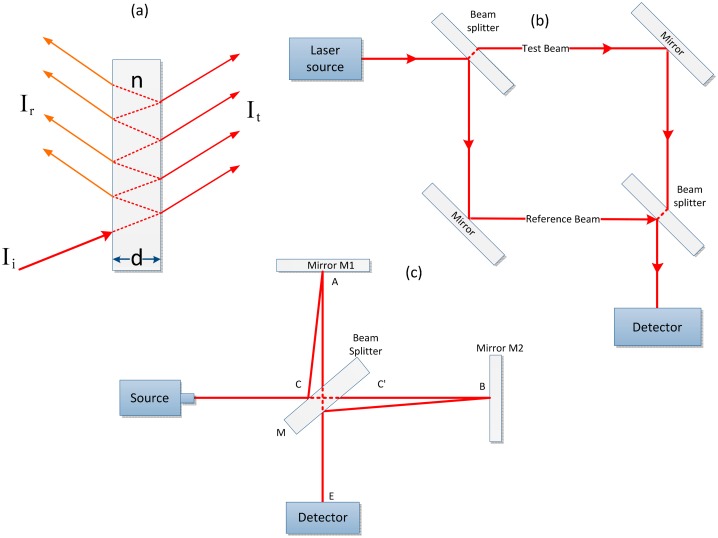
(**a**) FPI showing interference at the parallel planes of a plate with thickness d and n; (**b**) Mach-Zehnder interferometer (M = mirror and B = beamsplitter); (**c**) Path of light for a Michelson interferometer.

A FPI is highly sensitive with respect to geometric properties for which small changes can be measured [59,60]. The main advancement in the Fabry-Perot setup is a nanostructured FPI, which is more sensitive than a conventional FPI configuration [61]. The basic principles of operation of the device are shown in Figure 3a. Light enters the cavity and the nanopores in between the two plates, undergoes multiple internal reflections and causes interferences in the cavity and nanopores. Next, transducing signals are generated as reflected or transmitted interference fringes from the FPI. For this FPI device, the reflected signal is used as the sensing signal. If the pressure changes due to the presence of biochemicals, then the effective *n* will change inside the FPI cavity and nanopores, causing the reflected interference fringes to shift [62]. These sensitive parameters are used for various optical applications. A Fabry-Perot device can be used in a laser resonator to obtain single longitudinal modes. The most significant application of FPI is in high resolution optical spectroscopy. The geometric properties of FPI are related to the wavelength variations, and these variations can be adjusted using divergent light [56].

A MZI is a frequently used interferometer due to its flexibility, ease of use, ease of miniaturization, accessibility and appropriateness [63]. The MZI is a simple device for presenting interference using division of amplitude. First, a beamsplitter is used to split light from a light source into two components, which are recombined by a second beamsplitter. The relative phase difference is measured by the beam along the two paths, and the second beamsplitter will display the beam with an efficiency varying from 0% to 100% [61]. This device can be further miniaturized with integrated optics for the scheme shown in Figure 3b. In a Mach-Zehnder interferometer, the s-polarization light is used as a reference for certain applications. The s-polarization and p-polarization light beams are recombined to interfere and generate a set of interference fringes after passing over the sample arm. The interference fringe shows a path difference of 2π radians between the reference and the sample light beam. If there is an alteration in the sample value that causes a change, the phase shifts, and thus the position of the fringes shifts [53].

A Michelson interferometer consists of a monochromatic light source that strikes the beam splitter and is reflected back from two 100% mirrors (M1 & M2) that are placed 90° apart from the beam splitter, as shown in a Figure 3c. Because the beam splitter is partially silvered, one beam is reflected at nearly 90° at point C, and the second is transmitted through the beam splitter from point *C′*. After reflection from their respective mirrors, both beams are recombined by the beam splitter at a different point *C′*, and an interference pattern is visible through the detector to the observer at point *E*. The observed effects are identical to those formed by placing surfaces *B′* and *A* at point *E*. The path lengths are carefully equalized due to the short coherence length (micrometers) of the light requirement with the use of white light. The beam splitter simultaneously combines the effect of both the M1 and M2 reflected light beams to produce interference that can be determined by a detector [64].

## 4. SPR Phase Detection *via* Interferometry Schemes

To enhance the detection and sensing capability of an SPR sensor, interferometric techniques are used for SPR phase measurement. The SPR sensor sensitivity is increased in a precise manner when two beams are multiplexed via interferometry, and the information is extracted from the interference patterns to indicate the phase of the reflected light wave [65]. Three different interferometric techniques (FPI, MZI and Michelson interferometer) integrated with the SPR architecture for phase detection will be discussed. The phase SPR interferometry detection schemes are used to develop proper phase-sensitive SPR sensors with higher throughput and the ability to detect smaller molecules [66]. The optical scheme and electronics that define the minimal phase difference can be fixed using SPR interferometry techniques. The SPR interferometer provides spatial phase resolution that can consider the characteristics of the *n* distribution on the surface of the measureand [43]. Therefore, three different interferometry techniques are discussed for SPR phase detection together with applications.

### 4.1. SPR Phase Detection via Fabry-Perot Interferometer

**Figure 4.**
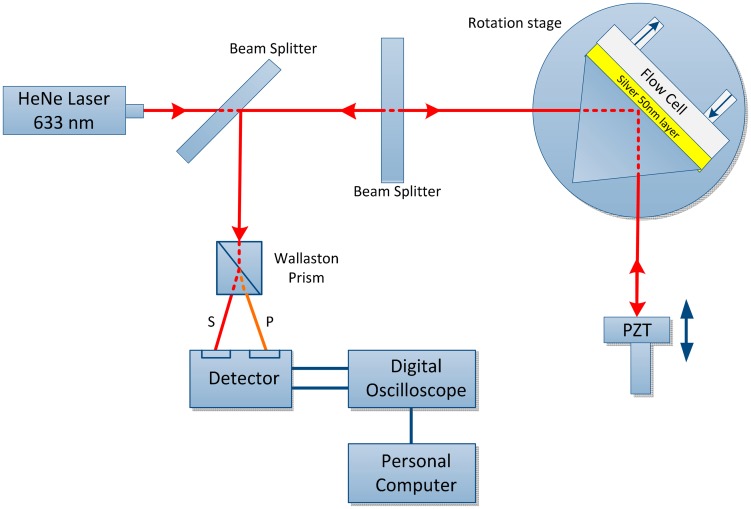
FPI optical configuration used to measure the differential SPR phase [67].

Recent progress towards a FPI for practical imaging is represented by a differential phase multi-pass SPR biosensor. This sensor consists of a multi-layer structure with improved algorithm. In recent years, the SPR sensors have achieved popularity among the biosensing community. The SPR phase is measured by a FPI in such way that the signal light passes through the sensor many times to obtain the SPR phase commutative effect, therefore amplifying the SPR phase [67]. The system is based on a differential SPR phase scheme incorporated into the FPI configuration as shown in Figure 4.

Experimental results acquired from protein-DNA and antibody-antigen binding reactions using a FPI with a contribution to the Michelson configurations indeed provide phase amplification factors of 2.26 and 2, respectively, compared with the conventional MZI configuration. Therefore, the sensitivity is enhanced, and the resolution is improved by a factor of at least two due to the phase amplification effect [68].

### 4.2. SPR Phase Detection via Mach-Zehnder Interferometer

**Figure 5.**
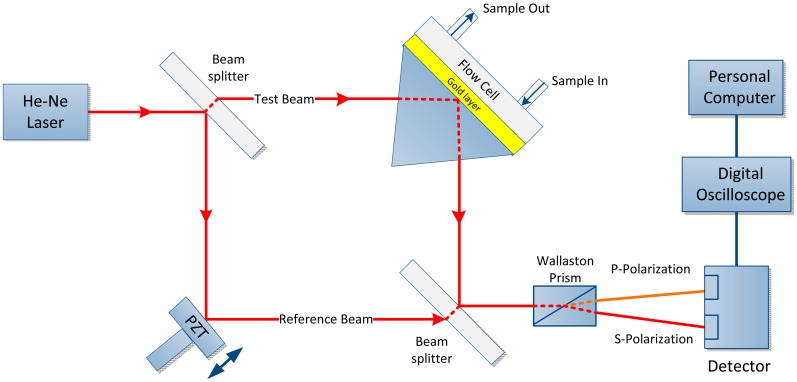
Experimental setup used to measurement the differential SPR phase shift representing s- and p-polarizations [69].

The main purpose of MZI to determine the phase changes when SPR occurs and also examine the threshold sensitivity of MZI as the *n* changes to Δ*n* [43]. A highly sensitive SPR biosensor is presented using the MZI design. The unique feature of this setup is the incorporation of a Wollaston prism that is used to interrogate the s- and p-polarizations simultaneously to measure the phase, as shown in Figure 5. Because SPR only affects the p-polarization, the s-polarization is employed as the reference signal. Therefore, the phase noise due to common path can be removed by taking the phase difference between the s- and p-polarization but retain the phase changes due to the SPR. A sensitivity of 5.5 × 10^−8^ refractive index units (RIU) can be obtained from this experimental setup using glycerin/water mixtures. A significant improvement can be achieved with the use of gold as the sensor surface. Such a sensitivity improvement can be highly useful in biological and chemical sensing and may enable potential replacement of conventional biosensing techniques [69].

**Figure 6.**
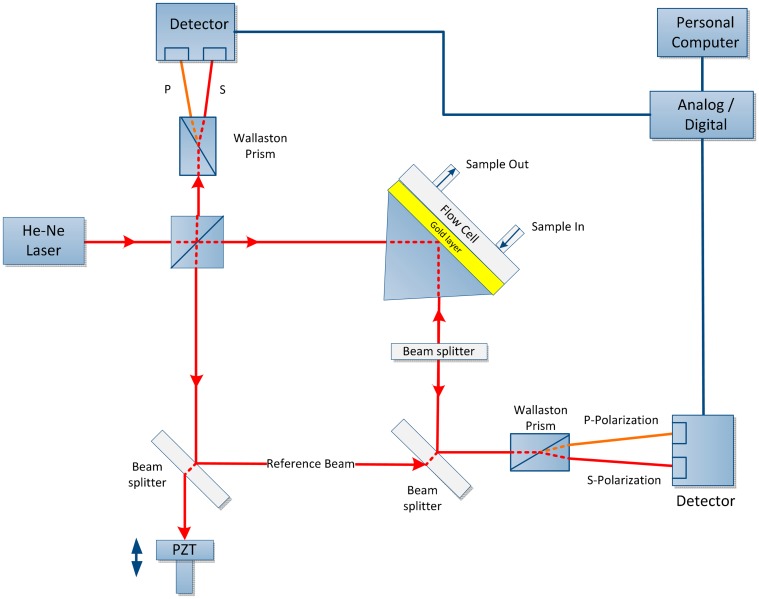
Experimental setup containing Mach-Zehnder and Michelson interferometers used to measure differential phase [70].

A double-pass SPR biosensor is a combination of MZI and Michelson interferometer with differential phase interrogation is a new scheme that provides enhanced sensitivity detection of up to a factor of two due to phase amplification effect, as shown in Figure 6. This approach enables an improvement in sensitivity compared with that of the conventional Mach-Zehnder setup without compromising the system complexity. This configuration is useful for detecting low-molecular-weight biomolecules [70].

The MZI can be miniaturized to form a nanostructured sensor with an ultra-compact size and high sensitivity. The sensor was assembled using a flexible nanofiber with a diameter of 700 nm. The detection *n* limit is nearly 1.8 × 10^−6^. Although the nanosensor construction is simple, low-cost and compact, it is highly sensitive [71].

#### SPR Phase Detection via Optical Heterodyne Interferometry

In SPR sensors, an amplitude measurement is collected in a straightforward manner because the photodetector directly determines the intensity. The direct phase measurement is difficult because due to high intensity oscillations of a light in the range of 10^14^ Hz that cannot be captured by the detectors. Therefore, low frequency signal can be produced and measured for the phase measurement by using a detector. The optical heterodyne technique is used for SPR phase measurement in sensors and is one of the earliest techniques. Two laser beams are generated with a minor difference in frequencies and positioned to produce interference, resulting in a beat frequency that is the result of frequency difference between the beams. The beat signal is compared with a reference signal to get the phase difference using a phase meter [66].

**Figure 7.**
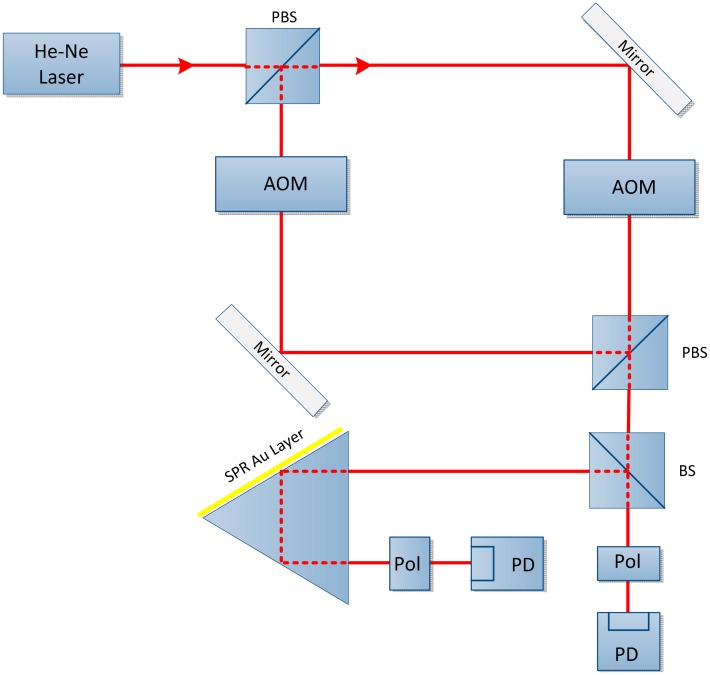
Schematic configuration diagram: M = mirror, PBS = polarizing beam splitter, BS = beam splitter, Pol = polarizer; AOM = Acousto-optomodulator, PhD = photo-detector (courtesy of [75]).

In 1996, Nelson and co. described an SPR phase sensor that used optical heterodyne detection. These researchers generated two light beams with a difference of 140 MHz using an acousto-optic modulator (AOM). The beam polarizing splitter was used to split the beam into a reference component and a signal component. The two components were rejoined with a help of linear polarizer and subsequently received by a photodetector as shown in Figure 7. A 140 MHz beat frequency signal was obtained by passing it through low-pass filter and oscillator was used to recombine the signal again at 140.01 MHz but for convenient manipulation, the frequency was reduced to 10 kHz. The two signal portions were subjected to the same procedure to incorporate a phase shift between the two polarizing component, except for passing through an SPR sensor. An accurate measurement of the SPR-induced phase shift was acquired by comparing the 10 kHz signal and the reference beams via the phase detection circuit. The optical heterodyne technique is insensitive to environmental noise and amplitude perturbations. The proposed setup was slightly complicated by limitations of the hardware due to unavailability of the proper frequency-shifting and phase detection instruments [72].

In 2001, Xing Long and his team built an immune sensor that based on the optical heterodyne technique. A transverse Zeeman laser was incorporated in the immune sensor with a minimum 30 kHz frequency difference in order to produce phase subdivision and improved resolution. The measurement accuracy for phase detection is enhanced by AC detection method for intensity instead of DC detection in order to avoid any shift [73]. A highly sensitive SPR biosensor was constructed using a same-path heterodyne interferometric system. A beam combiner was used to combine the He-Ne modulated s-wave and p-wave into a heterodyne light source with a frequency difference of 60 kHz. Use of the heterodyne interferometric common-path system to obtain the phase shift difference between the two waves offers the advantage of high sensitivity and real-time phase detection for monitoring of molecular interactions. This SPR biosensor was used to observe the interactions between sheep IgG covalently immobilized at the sensor chip surface and anti-sheep IgG in the running buffer [74].

A sensitive tunable optical heterodyne sensor, *i.e.*, a noise can be reduced significantly by using p- and s- polarizations from the surroundings. A quarter-wave-plate (QWP) is placed in front of and back of the sensor. The SPR sensor strongly absorbs the p-wave if the orientation of QWP is accurate. This effort provides the high sensitivity as well as the response curve optimization. With this new design, the sensor performance can be improved and it provides the highest sensitivity irrespective of the room temperature, and optimized thickness of the metal film and a reduced standard deviation [75].

### 4.3. SPR Phase Detection via Michelson Interferometer

A fiber-optic sensor based on a Michelson interferometer was developed to determine the liquid *n*. To fix the reference point, the fiber ends are dipped in the solution to have contact with the sample and the solvent, and the difference in interference fringes are used to find the n of the solution. The Michelson interferometer sensor offers a resolution of approximately 9.6 × 10^−6^ RIU in the measurement region and provides high resolution where the concentration of the solution is low. The sensor performance can be enhanced by increasing the fringe difference through use of a polarizer coupler instead of normal coupler [76].

**Figure 8.**
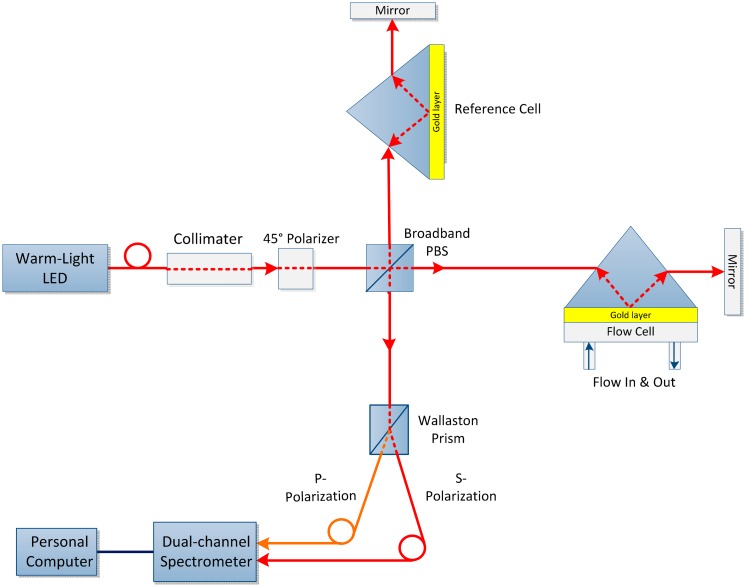
Schematic configuration for a differential spectral Michelson interferometer. NPBS denotes non-polarizing beam splitter [77].

This alteration results in a novel SPR biosensor with a wide dynamic range based on differential phase Michelson interferometry as shown in Figure 8. A white light source was utilized to measure the corresponding phase changes at the optimized value of a wavelength. Because the induced phase change is highly wavelength dependent with a fixed incident angle, ultra-high sensitivity can be achieved for phase-sensitive detection. This system provides an extended dynamic measurement range and optimal sensitivity while uncompromising the phase resolution, compared with that of existing laser-based phase detection setups [77]. The results obtained from sodium chloride solutions have 2.6 × 10^−7^ RIU detection limit with a *n* span of 10^−2^ RIU, that is comparatively broader than the current laser based method [78].

## 5. Interferometry Schemes and Trade-Offs

The sensitivity, fabrication, reproducibility, detection accuracy and operating range are the parameters that must be compared with those of other SPR interferometry schemes as summarized in Table 2. The best SPR sensor is one that combines a long operating range, the ability to reproduce results, ease of fabrication, and high detection accuracy and sensitivity [79].

The fabrication of an optical sensor consists of a FPI with better detection sensitivity of molecules that adsorbed on a gold surface. The SPR sensor with one reflective layer consists of a gold film with a local SPR. The sensor sensitivity can be further enhanced by depositing Au nanoparticles on the layer [80].

A new optical FP cavity technique that incorporates the SPR is examined theoretically. This improved and modified model of the FP has better impact on the phase response as compared to conventional phase-detection technique that is based on the Kretschmann configuration (MZI and Michelson interferometer). This high resolution FP cavity technique only requires the values of the power spectrum for the measurement of phase response over a narrow wavelength span. Therefore, this configuration is more attractive for highly sensitive sensing applications [81]. The prism-based SPR sensing arrangements (MZI and Michelson interferometer) have a number of disadvantages, such as the presence of several mechanical (moving) and optical components and bulky size. Furthermore, the prism-based SPR sensors cannot be used for remote sensing applications [79]. The FPI has certain drawbacks that restrict its use, and the main disadvantages of a Fabry-Perot are the highly complex and multiple steps involved in the fabrication process, low mechanical strength, surface imperfections affected by the removal of material and the need for special splicing programs; integration on a chip is also difficult due to its facets. Therefore, this setup requires a rather sophisticated fabrication tool and a sophisticated material growth procedure [82]. Another disadvantage is the low finesse intensity reflection [83,87]. Therefore, due to these problems, the overall system complexity is increased, and the performance and robustness of the interferometers are compromised [88].

Together with the increasingly fine and delicate nanoscale device manufacturing technologies, the accompanied sensing approaches are becoming more important for detection of small changes in the parameters used in the biochemical area. Several experimental schemes exist to measure the phase change of the SPR excitation, *i.e.*, phase shifting interferometry (PSI), which includes the Michelson & FPIs, and heterodyne interferometry (HI), which includes the MZI. Still, PSI suffers from certain disadvantages because it is not invulnerable to environmental noise, mechanical vibration and temperature with limited sensitivity. The HI is an optical modulation technique that yields higher sensitivity than PSI due to noise suppression from environmental disturbances, and the phase difference produced by the photodetector is amplified by a lock-in amplifier [84].

The Michelson interferometer is the most commonly used optical interferometry configuration. This instrument has a prime field of view for a specified wavelength, is relatively simple in operation and the tuning of wave plates can be carried out by mechanical rotation compared with the voltage control of Lithium Niobate optical modulators or piezoelectric crystals. However, Michelson interferometers have their own disadvantages; they have a restricted wavelength range, pre-filters are required (which restrict transmittance) and the conventional Michelson interferometer method has a narrow field of view [85].

## 6. Comparison of Phase with Angle, Wavelength and Intensity

**Figure 9.**
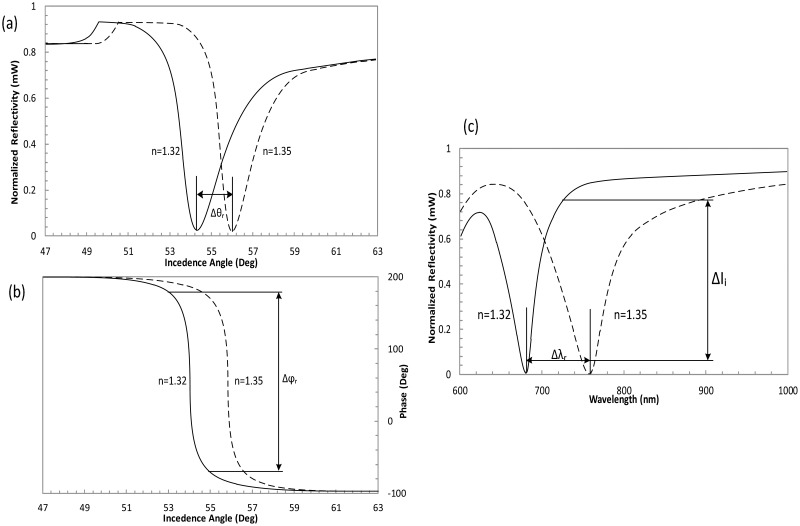
Light wave excitation of the SPW at the wavelength of 682 nm for reflectivity and phase in the Kretschmann configuration with respect to (**a**) an incidence angle of two different dielectrics; (**b**) a phase change of two different dielectrics; (**c**) the wavelength for two different dielectrics (angle of incidence = 54°).

SPR biosensors were investigated with an aim to produce a portable and cheap substitute to conventional setups for “real-time” analysis of complete cell-ligand interactions. Four approaches are used to measure SPR and their comparison is given in Table 3. The first approach is a wavelength-dependent SPR setup, *i.e.*, fiber optic based SPR biosensors, in which SPR sensor interrogates the trace on the basis of wavelength was developed to ensure continuous real-time data monitoring. The second approach is an integrated angle-based SPR biosensor with reflecto-meter that was improved to facilitate the biosensing community and its applications [89]. The third approach is an intensity measurement for SPR corresponding to *n* change with fixed values of angle and wavelength [90]. The last approach consists of a highly sensitive phase detection method used to determine the phase change with respect to changes in *n* of the sensing material compared with the wavelength, intensity and angle parameters [91–93]. In SPR experimental setup using Kretschmann configuration for angle modulation, the Δ*θ_r_* is the change in the incidence angle of the reflected light at fixed wavelength for two different *n* of the dielectric (*n* = 1.32 and *n* = 1.35). The change in phase Δ*ϕ_r_* of the reflected light when using phase modulation at fixed wavelength and angle of incidence, Δ*l_i_* is the change in the reflected light intensity at fixed value of an incidence angle and the wavelength, for wavelength modulation technique the Δ*λ_r_* is the change at fixed angle of incidence for SF14 glass prism with 50 nm gold film as depicted by Figure 9 [9]. The dynamic range of the phase measurements has a much stronger response to the *n* change compared with that of the intensity-sensitive SPR [94]. Therefore, the sensitivity is at least 50 times greater than that of the conventional intensity-based SPR configurations [95]. The detection limit of the phase SPR sensor is 100 times lower than the intensity interrogation [96]. The result from the interferogram shows that the phase of the beam has a notably steep slope at the resonance angle compared with that of intensity sensing [97]. It is observed that the phase can change more abruptly than the intensity of the thickness of thin metal film or the *n* of the media on the sensor surface, and therefore, this method has become an attractive sensing technology [84].

The high sensitivity of the SPR phase detection method is obtained from the resonant dependency on the parameters of a laser light reflected from a metal surface, as a result of changes in the *n* of the adjacent medium [43,98]. Furthermore, phase resolution provides the possibility of examination of a medium with the use of a thin film [99]. Therefore, the latest research tell us that SPR phase has the ability to attain a lower detection limit and higher efficiency [100]. In this paper, we have reviewed various SPR phase detection schemes and configurations, especially phase detection via interferometry.

## 7. Applications of SPR Interferometry

In recent years, SPR interferometry has been increasingly used in the field of biological and chemical sensing and has replaced the traditional methods. The SPR interferometry is particularly suitable for measurement of interaction partners, *i.e.*, nucleic acids [101], cells [102], proteins [103], antigens [104] and hormones [105]. For example, in chemical sensing, an impurity of glycerol in water with a level of 10 percent of volume was determined, which relates to a resolution of 10 RIU. This value is the minimum concentration level that can be determined by relative changes in the interference patterns [106]. The surface roughness of several dielectric materials with different deposition methods were determined via an SPR interferometer in the visible near infrared region (NIR). The dielectric values and the surface roughness at several deposition rates of thin Au film were also determined [65].

In biological sensing, SPR interferometry has made it possible to monitor the binding kinetics in less than 1300 spots in a protein microarray with a detection limit of ∼0.3 ng/cm^2^ and a 1 s time resolution. Such a setup should capture the high-throughput binding kinetics of small (∼200 Da) ligands on large protein microarrays and provide the absolute binding stoichiometry and bound amount as well [14]. The SPR microscopy setup dramatically decreases the appearance of speckles by producing high quality images of protein spots on the Au surface. The image size was 1280 × 1024 with a 18 × 18 pixel area selected for image analysis [107]. The SPR approach is recommended for initial kinetic measurements due to the spot sensing ability. This system is used to adsorb lipase from Thermomyces lanuginosus on a surface. The adsorption saturates at 1.30–1.35 mg/m^2^ with Thermomyces lanuginosus concentrations of 1000 nM [108]. Novel SPR biosensors based on Ag-graphene nanohybrids and Au-graphene nanohybrids were developed for the detection of mouse IgG. The SPR biosensors based on Ag-graphene nanohybrids and Au-graphene nanohybrids show good results with mouse IgG in a concentration range of 0.15–40.00 μg·mL^−1^ and 0.30–40.00 μg·mL^−1^, respectively [109].

## 8. Conclusions

This review paper provides an overview of the recent development in SPR sensors using interferometry and phase detection technique as well as certain drawbacks of the interferometry schemes. Additionally, certain solutions intended to increase the overall performance of SPR interferometry type sensors are covered.

Improving the performance of the SPR sensor is the main objectives in development for clinical applications. The theory shows that the performance of an SPR sensor depends predominantly on the noise properties of the detector and light source [110]. The sensitivity of a SPR biosensor is a fundamental feature for such applications. An improvement in sensitivity can be achieved if gold nanoparticles are exploited to functionalize the interacting surface. The gold nanoparticles are immobilized on a gold thin film surface of the SPR sensor [111–113]. The sensitivity enhancement of a SPR biosensor that includes a graphene layer on top of the gold layer has been demonstrated experimentally [114]. The performance of the SPR graphene biosensor was numerically and theoretically examined in terms of adsorption efficiency and sensitivity under changing conditions, including the number of graphene layers, the thickness of the biomolecule layer and the operating wavelength [115]. The utility of different bimetallic nanoparticle alloy combinations in SPR sensors based on this scheme is investigated in different approaches [116,117]. Silver, gold, copper and aluminum are selected for this analysis. The performances of the SPR sensors with different layers of bimetals or combinations of nanoparticle alloy are evaluated. The performance can be evaluated in comparison to three parameters: SNR, sensitivity and sensing layer operating range with respect to *n* values. The best bimetallic alloy combinations can be selected based on comparison and the selected logical criteria. The higher values of sensitivity, SNR and operating range can be achieved by using combination of bimetallic nanoparticle alloy [50]. The SPR phase technique is very popular measurement technique that can be modified further by adjusting the incident angle as indicated by a simulation and white Gaussian-beam light as a super continuum light source is used instead of LED light. Additionally, the gold-silver combination can be used for sensing so that the detected spectral phase signal can be significantly improved [78].

## Figures and Tables

**Table 1.** Description of different performance parameters.

**Performance Parameters**	**Description**
Sensitivity	The ratio of the change in the sensor output (y) (e.g., wavelength, intensity, angle of incidence, phase, and polarization of a light that interact with a surface plasmon wave) with respect to the change in *n* of the medium (e.g., analyte concentration) [49]. *S* = Δ*n*/Δ*y*
SNR	The ratio between the output intensity (*P_O_*) of the light to the input intensity (*P_I_*) of the light beam, and a high value of SNR is preferred for better results [31]. *SNR* = *P_O_/P_I_*
Accuracy	The degree of the sensor output provides the true value of the quantity to be measured (analyte concentration) [50].
Precision	The manner in which the results give the same reading when the repetitive measurements are carried out without any reference to the true value [51,52].
Repeatability	The capability of a sensor to reproduce same output over a certain interval of time while operating under a constant environmental condition [9].
Minimum Detection Limit	The lowest concentration level of an analyte that can be determined by the sensor [53].

**Table 2.** Tradeoffs in the Fabry-Perot, Mach-Zehnder and Michelson interferometers.

**SPR Interferometr**	**Fabry-Perot [67,80–83]**	**Mach-Zehnder** [43,69,84]	**Michelson** [78,79,85,86]
Advantages	High sensitivity, small size, high resolution.	Simple setup, good sensitivity, can be modified by adding other components.	Simple in operation and tuning, good sensitivity
Disadvantages	Highly complex fabrication, low mechanical strength, temperature limitation, low finesse intensity reflection.	Bulky size, moving mechanical parts, high cost.	Restricted wavelength range, required pre-filters, environmental noise, and mechanical vibrations.
Sensitivity	2.3 × 10^−8^ RIU	5.5 × 10^−8^ RIU	2.2 × 10^−7^ RIU
System Size	Small	Medium-Large	Medium
Applications	SPR intensity image of the 15 sensor sites covered by different salt water mixture. The Ag, BaTiO_3_, and Au layers are deposited on a quartz substrate to develop the Fabry-Perot sensor for measuring 1,6-hexanedithiol on the Au surface.	Measuring of the bovine serum albumin (BSA) binding reaction with its antibodies by SPR Mach-Zehnder Setup. The MZI setup is used to determine significant phase changes by replacing the 100% gas to 100% Ar gas.	The the addition of 10% NaCl solution into the pure water measured by SPR Michelson interferometer.

**Table 3.** Comparison of intensity, angle, wavelength and phase detection with respect to three parameters [66].

**SPR Schemes**	**Intensity**	**Angular Interrogation**	**Wavelength Interrogation**	**Phase Detection**
Resolution	10^−5^ RIU	5 × 10^−5^ RIU	10^−6^ RIU	4 × 10^−8^ RIU
Dynamic range	0.05 RIU	0.1 RIU	>0.1 RIU	5 × 10^−4^ RIU
SPR imaging	Convenient	Difficult	Difficult	Convenient
